# Prediction of prostate cancer grade using fractal analysis of perfusion MRI: retrospective proof-of-principle study

**DOI:** 10.1007/s00330-021-08394-8

**Published:** 2021-12-16

**Authors:** Florian Michallek, Henkjan Huisman, Bernd Hamm, Sefer Elezkurtaj, Andreas Maxeiner, Marc Dewey

**Affiliations:** 1 Department of Radiology, Charité – Universitätsmedizin Berlin, corporate member of Freie Universität Berlin, Humboldt-Universität zu Berlin, and Berlin Institute of Health, Charitéplatz 1, 10117 Berlin, Germany; 2 Department of Radiology, Radboud University Nijmegen Medical Centre, Nijmegen, The Netherlands; 3 Institute of Pathology, Charité – Universitätsmedizin Berlin, corporate member of Freie Universität Berlin, Humboldt-Universität zu Berlin, and Berlin Institute of Health, Berlin, Germany; 4 Department of Urology, Charité – Universitätsmedizin Berlin, corporate member of Freie Universität Berlin, Humboldt-Universität zu Berlin, and Berlin Institute of Health, Berlin, Germany

**Keywords:** Prostatic neoplasms, Neoplasm grading, Perfusion, Fractals, Multiparametric magnetic resonance imaging

## Abstract

**Objectives:**

Multiparametric MRI has high diagnostic accuracy for detecting prostate cancer, but non-invasive prediction of tumor grade remains challenging. Characterizing tumor perfusion by exploiting the fractal nature of vascular anatomy might elucidate the aggressive potential of a tumor. This study introduces the concept of fractal analysis for characterizing prostate cancer perfusion and reports about its usefulness for non-invasive prediction of tumor grade.

**Methods:**

We retrospectively analyzed the openly available PROSTATEx dataset with 112 cancer foci in 99 patients. In all patients, histological grading groups specified by the International Society of Urological Pathology (ISUP) were obtained from in-bore MRI-guided biopsy. Fractal analysis of dynamic contrast-enhanced perfusion MRI sequences was performed, yielding fractal dimension (FD) as quantitative descriptor. Two-class and multiclass diagnostic accuracy was analyzed using area under the curve (AUC) receiver operating characteristic analysis, and optimal FD cutoffs were established. Additionally, we compared fractal analysis to conventional apparent diffusion coefficient (ADC) measurements.

**Results:**

Fractal analysis of perfusion allowed accurate differentiation of non-significant (group 1) and clinically significant (groups 2–5) cancer with a sensitivity of 91% (confidence interval [CI]: 83–96%) and a specificity of 86% (CI: 73–94%). FD correlated linearly with ISUP groups (*r*^2^ = 0.874, *p* < 0.001). Significant groupwise differences were obtained between low, intermediate, and high ISUP group 1–4 (*p* ≤ 0.001) but not group 5 tumors. Fractal analysis of perfusion was significantly more reliable than ADC in predicting non-significant and clinically significant cancer (AUC_FD_ = 0.97 versus AUC_ADC_ = 0.77, *p* < 0.001).

**Conclusion:**

Fractal analysis of perfusion MRI accurately predicts prostate cancer grading in low-, intermediate-, and high-, but not highest-grade, tumors.

**Key Points:**

*• In 112 prostate carcinomas, fractal analysis of MR perfusion imaging accurately differentiated low-, intermediate-, and high-grade cancer (ISUP grade groups 1–4)*.

*• Fractal analysis detected clinically significant prostate cancer with a sensitivity of 91% (83–96%) and a specificity of 86% (73–94%)*.

*• Fractal dimension of perfusion at the tumor margin may provide an imaging biomarker to predict prostate cancer grading*.

**Supplementary Information:**

The online version contains supplementary material available at 10.1007/s00330-021-08394-8.

## Introduction

Angiogenesis is a hallmark of cancer and is closely intertwined with tumor development and metabolism [1, 2]. The dedifferentiation of tumor tissue is related to an “angiogenic switch” and ensuing changes in vascular architecture [3]. Such phenotypes of tumor microvascularization have been visualized, e.g., using contrast-enhanced ultrasound microscopy [4]. Compared with the normal vascular architecture in the human body, changes in tumor vascular architecture in early stages of the dedifferentiation sequence match the alimentary need of the growing tumor. In general, blood vessel trees follow physiology-determined branching rules over a multitude of scales. This so-called scale invariance is a central characteristic of fractals. Fractals can be found everywhere in nature and are a fundamental principle of biological structure and function. Perfusion is a case in point—it is a physiological process featuring a fractal organization [5, 6], which can be unraveled by fractal analysis based on radiological and nuclear medicine imaging methods [7, 8]. Fractal analysis can measure an object’s geometrical complexity, or chaos, which can be quantified in terms of fractal dimension (FD). Since fractal analysis is based on pathophysiological principles of perfusion, it can be expected to reveal information on the underlying biological correlate of perfusion abnormalities. The pivotal role of angiogenesis especially in prostate cancer (PCa) has been acknowledged [9, 10] and histological analysis methods have been debated [11]. However, the role of perfusion—as the functional correlate to vascular anatomy—is currently of minor priority for clinical imaging workup in patients with PCa.

The grading of PCa has decisive relevance for clinical management of patients and prognosis. The standard for diagnosis of PCa is biopsy with histological grading according to the Gleason grading system [12]. In 2016, the International Society of Urological Pathology (ISUP) suggested grade groups ranging from 1 to 5 [13, 14] for the classification of tumors as a more comprehensive grade reporting system [15]. Importantly from a clinical perspective, patients with ISUP grade group 1 lesions and some patients with ISUP grade group 2 lesions and a low percentage of Gleason score 4 can be considered for active surveillance [16]. Multiparametric magnetic resonance imaging (mpMRI) of PCa at a magnetic field strength of 3 T has been implemented and has excellent sensitivity and negative predictive values in detecting PCa [17, 18], including the transition zone [19], and helps to preoperatively predict clinically significant cancer [20]. Clinically, the scope of prostate MRI is mainly focused on cancer detection by implementing the Prostate Imaging-Reporting and Data System (PI-RADS) in its current version 2.1 [21] with a trend towards non-contrast biparametric protocols by omitting dynamic contrast-enhanced (DCE) sequences [22]. Indeed, tracer kinetic parameters obtained from perfusion MRI have shown potential to assess the aggressiveness of PCa in the peripheral zone [23]. Moreover, analysis of apparent diffusion coefficient in MRI has shown some relation to tumor grade [24, 25]. However, no method for non-invasive tumor grade prediction is available to match the clinically needed accuracy.

**Fig. 1:**
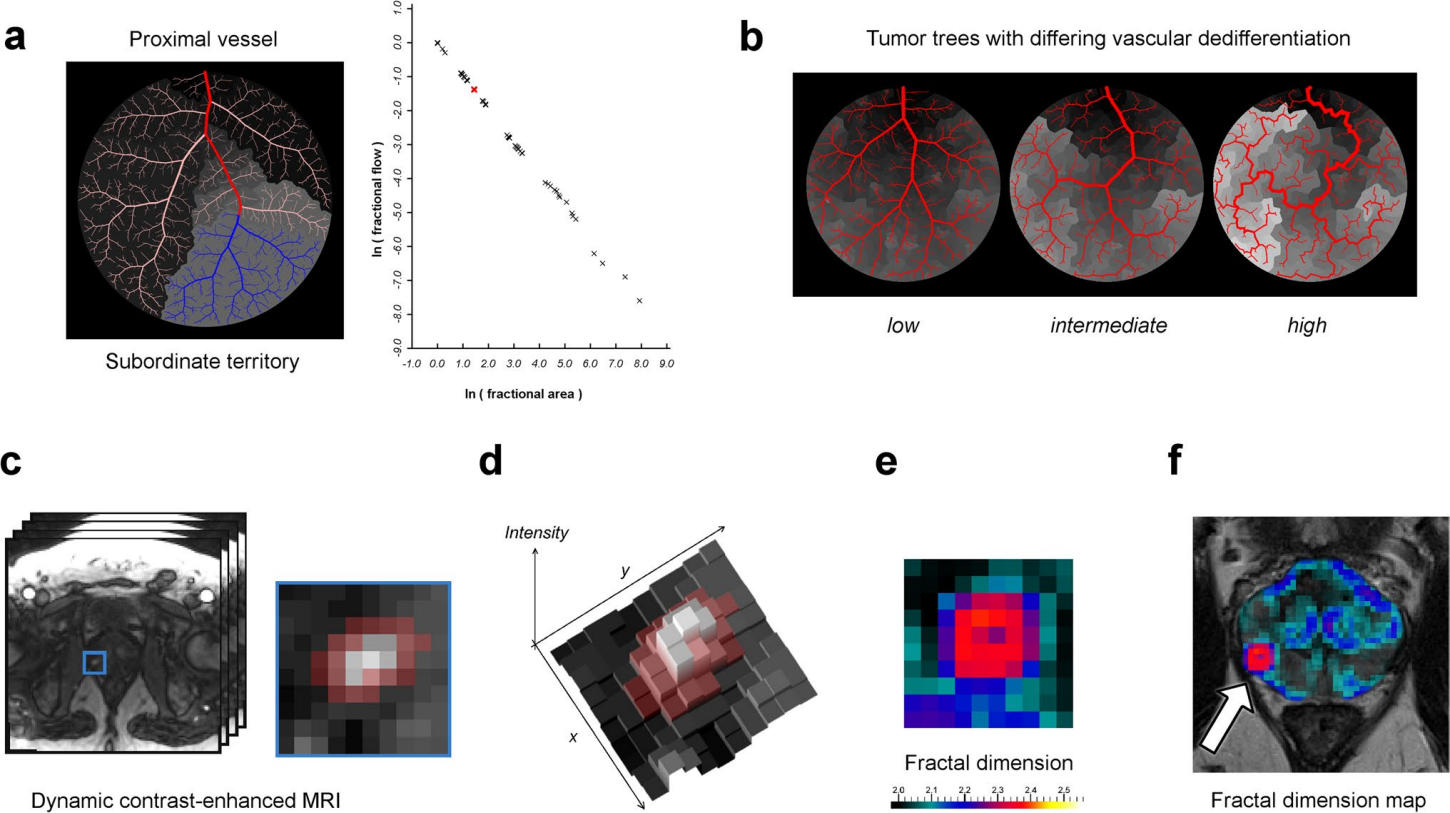
Hypothesis and rationale for fractal analysis of perfusion territories. **a** Perfusion territories exist for each vascular scale and include a proximal, regulating feeding vessel (red), depending distal vessels (blue), and the corresponding perfused tissue (gray area). A fractal relationship exists between flow and size of the territory; see Supplementary Fig. S1 for an animated version. **b** Vascular dedifferentiation during tumor angiogenesis alters the perfusion pattern, which is depicted in gray levels underneath the trees. **c**–**f** Illustration of fractal analysis. **c** Perfusion MRI with the tumor area being marked and magnified (blue frame). Pixels marked in red indicate the tumor margin and adjacent tissue and constitute the pathophysiologically relevant region of interest. **d** To calculate fractal dimension, the image is considered a texture embedded in two-dimensional space with intensity as third dimension. **e** Fractal dimension map of the tumor area and (**f**) of the whole prostate with the corresponding T2-weighted image underneath. This example shows a prostate cancer focus (arrow in **f**) in the right midglandular peripheral zone. The tumor margin has a mean local fractal dimension of 2.344, which corresponds to ISUP grade group 3 and was confirmed histologically

Our study explores the potential of fractal analysis of perfusion as a surrogate for tumor vessel dedifferentiation. We evaluate fractal dimension (FD) as a quantitate biomarker obtained from clinical routine DCE-MRI sequences for tumor grade prediction in PCa. The hypothesis of this work is as follows (see Fig. 1): (I) The organization of blood vessel trees entails fractal perfusion territories, which determine the observable perfusion pattern (Fig. 1a). (II) Alteration of vascular organization and perfusion territories, e.g., by tumor angiogenesis, can be measured by fractal analysis (Fig. 1b). (III) Fractal analysis of perfusion can be used to discriminate individual PCa grades using clinical perfusion MR imaging data (Fig. 1c–f) and outperforms the current research standard, i.e., ADC measurement.

## Materials and methods

### Investigative steps

To test the hypothesis of this study, three investigative steps are performed: (I) A mathematical formulation of fractal perfusion territories justifying fractal analysis is proposed. (II) In silico experiments are conducted to validate the application of fractal analysis under varying vascular conditions. (III) Fractal analysis is applied in vivo to clinical prostate mpMRI for non-invasive prediction of tumor aggressiveness using an openly available dataset with clinical routine imaging and histology data.

### Significance of fractal dimension

Fractal dimension (FD) can be interpreted as a measure of chaos or roughness. Consider a sheet of paper, which is a two-dimensional object when its thickness is neglected. When the sheet of paper is crumpled up, it occupies a certain volume with the actual volume changing according to how much it is crumpled. FD provides us with a measure of roughness, which reflects the amount of crumpling in this example. Consequently, the dimensionality of the sheet, or its FD, exceeds its topological dimension of two and is capped by the embedding dimension of three with its actual value in between. Here, FD thus ranges from two to three and can be interpreted as a measure of correlation. More specifically, FD is measured in terms of feature propagation over scale with a bi-logarithmic linear regression from observed feature against scale [7]. An object is a fractal if it features scale-invariant self-similarity, which can be determined from that regression, and can be found in many biological structures such as the vascular tree [26].

Two objects with a similar fractal dimension do not necessarily resemble each other. Rather, FD can be considered a descriptor of the object’s geometrical complexity. In medical imaging, data can be represented in topologically two-dimensional grayscale images. These images can be interpreted as textures or terrain maps with intensity representing the texture’s height (Fig. 1). Thus, such images can be assigned a topological dimension of two and an embedding dimension of three similar to a crumpled sheet of paper. In the context of textures, an intensity distribution with a high spatial correlation tends towards integer values of FD (i.e., near 2.0 or 3.0), whereas randomly distributed intensity variations tend towards an FD of 2.5. As a subclass of texture analysis methods, fractal analysis constitutes a quantitative descriptor of chaos found in textures. When applied to DCE perfusion images, fractal analysis relates the phenotypical appearance of the perfusion imaging texture to its underlying biological correlate, i.e., vasculature. Therefore, FD as a quantitative texture analysis feature yields pathophysiologically meaningful information on perfusion and, thereby, vascular structure.

### Pathophysiological hypothesis

Our pathophysiological hypothesis for fractal analysis in PCa perfusion imaging is based on perfusion regulation, which takes place over a large range of invariant and self-similar vascular scales, mainly ranging from the small arteries to the precapillary arterioles. Dilation and constriction of a vascular segment governs blood supply to the depending microcirculation, i.e., the perfusion territory. The superposition of these territories results in a macroscopically observable perfusion pattern, which reflects the fractal properties of the underlying vascular tree. Angiogenesis mainly takes place in the tumor margin, and vessel architecture stabilizes in the tumor center [27]. Thus, the perfusion pattern of the tumor margin might provide information on the dynamic stage of tumor development when compared to the perfusion pattern of adjacent host tissue. The fractal organization of blood vessel trees applies to the anatomically deduced perfusion pattern and constitutes the fractal feature, which is measured over scale during fractal analysis [26]. Alteration of vascular organization and perfusion territories, which is related to vascular dedifferentiation in tumor angiogenesis, can thus be measured by fractal analysis at the interface region between tumor and host tissue, i.e., the tumor margin. In summary, FD is interpreted as a bulk measure of geometrical complexity found in the perfusion pattern of the viable, hyperperfused margin of the tumor and is assumed to provide information on the underlying vascular dedifferentiation. Therefore, FD is hypothesized to enable prediction of tumor grade in PCa studied from perfusion imaging data.

### Mathematical formulation of perfusion territories

To better understand the organization of PCa perfusion, we deduced an algebraic concept based on physiological consideration, which can be found in the Supplementary methods. This formulation is part of the results of this study and constitutes the basis for the further experiments.

### In silico* experiments*

**Fig. 2:**
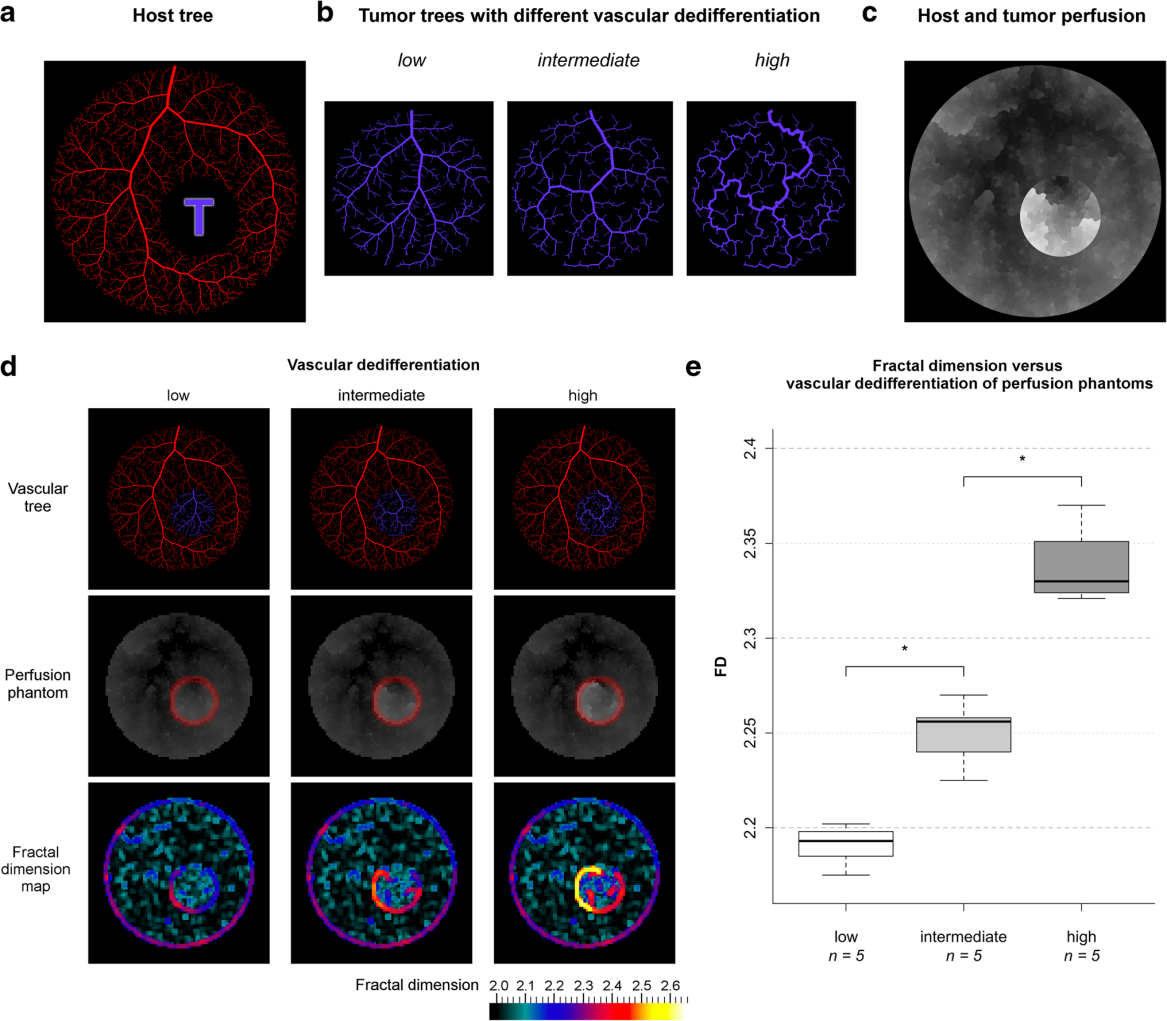
Results of in silico experiments. **a** Host vessel tree (red) with a placeholder for later insertion of a tumor tree (marked with a purple T). **b** Tumor trees (purple) with varying optimization targets to represent different stages of vascular dedifferentiation: intravascular volume (low), endothelial surface (intermediate), and vessel length (high). **c** Perfusion territories resulting from host and tumor tree anatomy. The gray value is additively proportional to the quotient of ln *Q*_frac_ / ln *a*_frac_ multiplied with the respective perfusion rate at each vascular scale according to the definition of the vascular model (here rescaled for visual purposes). **d** From top to bottom: complete vascular phantom for each dedifferentiation stage after inserting tumor trees (purple) into the placeholders of the host trees (red). Perfusion phantoms were calculated (original scale). The tumor margin is marked in red. Maps of the local fractal dimension (FD) were generated. **e** Boxplot of FD against tumor vascular dedifferentiation stage. Significances of groupwise differences are indicated by asterisks: *—*p* < 0.03; *n*—sample size per dedifferentiation stage

Based on our mathematical formulation, we generated in silico perfusion phantoms. We simulated ground-truth in terms of three well-defined vascular stages and applied fractal analysis to our phantoms to prove our concept. This approach allowed systematic alteration of experimental conditions of tumor perfusion territories. We used the constructive constrained optimization (CCO) algorithm [28] to generate in silico vascular trees: First, we simulated normal vascular trees of non-tumorous prostate tissue in a circular shape with a radius of 5 cm, leaving a placeholder for insertion of the tumor tree (Fig. 2a). Second, we generated tumor trees in varying dedifferentiation stages (low, intermediate, high) in circular shapes with a radius of 1.6 cm as explained in the Supplemental material (Fig. 2b). Those tumor trees were inserted into the placeholder of the normal tree. Subsequently, we calculated the perfusion territories from those vascular trees (animated Supplementary Fig. S1) and simulated contrast enhancement as surrogate for perfusion by downscaling to a typical clinical resolution for DCE images of 1.5 mm per pixel (Fig. 2c). All details of the in silico simulations are given in the Supplementary methods. Subsequently, we used fractal analysis to calculate local FD maps as specified in the Supplementary methods. Finally, we delineated the tumor-host interface region (marked in red, Fig. 2d) to measure the mean FD, which was hypothesized to correlate with simulated tumor grade.

### Patients and imaging dataset

For the in vivo experiments, we used the imaging dataset from patients of the PROSTATEx challenge [29, 30]. This dataset has been published under the Creative Commons Attribution 3.0 Unported License and the dataset remained unchanged. Institutional Review Board approval has been obtained by the providers of the dataset. The dataset was acquired as a consecutive series of routine patients. We included the available 112 prostate carcinomas with revealed ISUP grade groups obtained from in-bore MRI-guided biopsy in 99 patients, which were consecutively analyzed with blinding to the grade groups. Details on imaging parameters, employed drugs including contrast agents and scanners can be found in [29] and imaging sequences were compliant with the ESUR prostate MR guidelines [31]. In summary, imaging in patients was performed using two different MRI scanners from the same manufacturer with a magnetic field strength of 3 T. Imaging sequences included T2-weighted, proton density-weighted, dynamic contrast-enhanced (DCE), and diffusion-weighted imaging with mapping of apparent diffusion coefficients (ADC). DCE sequences were acquired using a 3-D turbo flash gradient echo sequence (in-plane resolution around 1.5 mm, slice thickness 4 mm, temporal resolution 3.5 s) while intravenous administration of a gadolinium-based contrast agent. The exact location of PCa in all MRI sequences is given as a coordinate of the lesion’s centroid. Histological grading in terms of the ISUP grade group is available for each individual lesion. Data used in this research were obtained from The Cancer Imaging Archive (TCIA) sponsored by the international society for optics and photonics (SPIE), National Cancer Institute/National Institute of Health (NCI/NIH), American Association of Physicists in Medicine (AAPM), and Radboud University [32].

### In vivo* image processing and fractal analysis*

All timepoints from the clinical routine DCE imaging sequence were subjected to fractal analysis of perfusion, and the analysis procedure was performed independently by one blinded reader (6 years of experience in urogenital imaging) and in *n* = 50 lesions by a second blinded reader (over 15 years of experience in urogenital imaging) to determine interobserver variability. First, we applied an individual noise-level adapted denoising scheme and intensity calibration as explained in the Supplementary material to ensure standardization of the imaging data. Second, a semi-automatic segmentation procedure was applied by fitting a serpentine-like ROI to the contrast-enhancing lesion periphery. Third, local FD maps were automatically calculated based on the blanket method [33] for each timepoint. The ROI with the maximum FD was subjected to statistical analysis. All image processing and the implementation of fractal analysis based on the DCE image texture is detailed in the Supplementary methods.

### In vivo* ADC measurements*

ADC was measured in the tumor region with the lowest ADC values, and the 25th percentile was subjected for statistical analysis. We adopted this approach from several reports [29, 34–36], which indicated that the use of a low percentile (in some studies even the 10th percentile) improves the correlation of ADC with Gleason grade.

### Statistical analysis

According to the pathophysiological hypothesis, a correlation between the highest mean FD of each lesion and its corresponding ISUP grade group was postulated. The Kruskal–Wallis test and pairwise group comparisons using Mann–Whitney *U* test with Bonferroni correction for multiple testing were performed. A significance level lower than 0.05 was considered significant, and adjusted *p* values with Bonferroni correction are reported. Correlation between FD and ISUP grade groups was evaluated by linear modeling. Interobserver variability of fractal analysis between two separate blinded readers (6 and over 15 years of experience in urogenital imaging) was evaluated using Cohen’s *κ* and Bland–Altman analysis in *n* = 50 cases. Diagnostic accuracy was analyzed using receiver-operating characteristic (ROC) analysis, and the area under respective ROC curves (AUC) along with sensitivity and specificity were calculated. To analyze the diagnostic accuracy of fractal analysis for two-class differentiation, i.e., differentiation of lower- and higher-grade lesions, the dataset was dichotomized according to each lesion’s ISUP grade group assignment at different group thresholds (i.e., pooled evaluation of group 1 versus 2–5, groups 1–2 versus 3–5, groups 1–3 versus 4–5, groups 1–4 versus 5). For analysis of diagnostic accuracy in all five grade groups, the multiclass AUC was calculated and its confidence intervals were computed by bootstrap as explained in [37]. As criterion for the selection of FD cutoffs for grade group differentiation, we used the efficiency or proportion of correctly classified observations which corresponds to the cutoff maximizing the efficiency formula *Ef* (*c*) = *pSe*(*c*) + (1 − *p*)*Sp*(*c*) where *Se* stands for sensitivity, *Sp* for specificity, and *p* for the estimated prevalence in the population (based on our sample), as reported in [38]. Statistical analysis was performed with R (v3.4.1; 30 June 2017, R Foundation for Statistical Computing).

## Results

### Mathematical formulation

Based on our mathematical assumptions, we found a fractal relationship between blood flow and perfused tissue area in the form of ${A}_{\mathrm{perf}}^{-FD}\propto {Q}_{\mathrm{perf}}$, which represents a power law scaling between the proximal, regulating part of the vascular tree and the distal, regulated tissue portion, i.e., the perfusion territory. *A*_perf_ is the size of a perfusion territory and *Q*_perf_ is the respective flow. FD is fractal dimension, which quantitatively characterizes the fractal relationship (Fig. 1a and animated Supplementary Fig. S1). This formulation demonstrates the fractal properties of perfusion territories, i.e., self-similarity and scale invariance.

### In silico* experiments*

To validate our mathematical formulation, we simulated perfusion using vascular in silico phantoms and tested whether fractal analysis correctly characterized the underlying vascular dedifferentiation stages. Examples of the three distinct simulated tumor grades are shown in Fig. 2a–d together with the corresponding perfusion simulation and FD map (all phantoms are included in the Supplementary Figs. S2-4). A total of 15 phantoms were analyzed with five samples per stage being modeled.

As shown in Fig. 2e, the median FD of the tumor margin increased with the dedifferentiation stage: median FD and interquartile range (IQR) was 2.193 (0.013), 2.256 (0.018), and 2.330 (0.027) in trees representing low, intermediate, and high stages of vascular dedifferentiation, respectively. Pairwise multiple group comparisons showed significant differences in FD (*p* < 0.03) among all three vascular dedifferentiation stages. FD correlated excellently with dedifferentiation stage (Spearman’s *ρ* = 0.94, *p* < 0.001).

### In vivo* experiments*

**Fig. 3:**
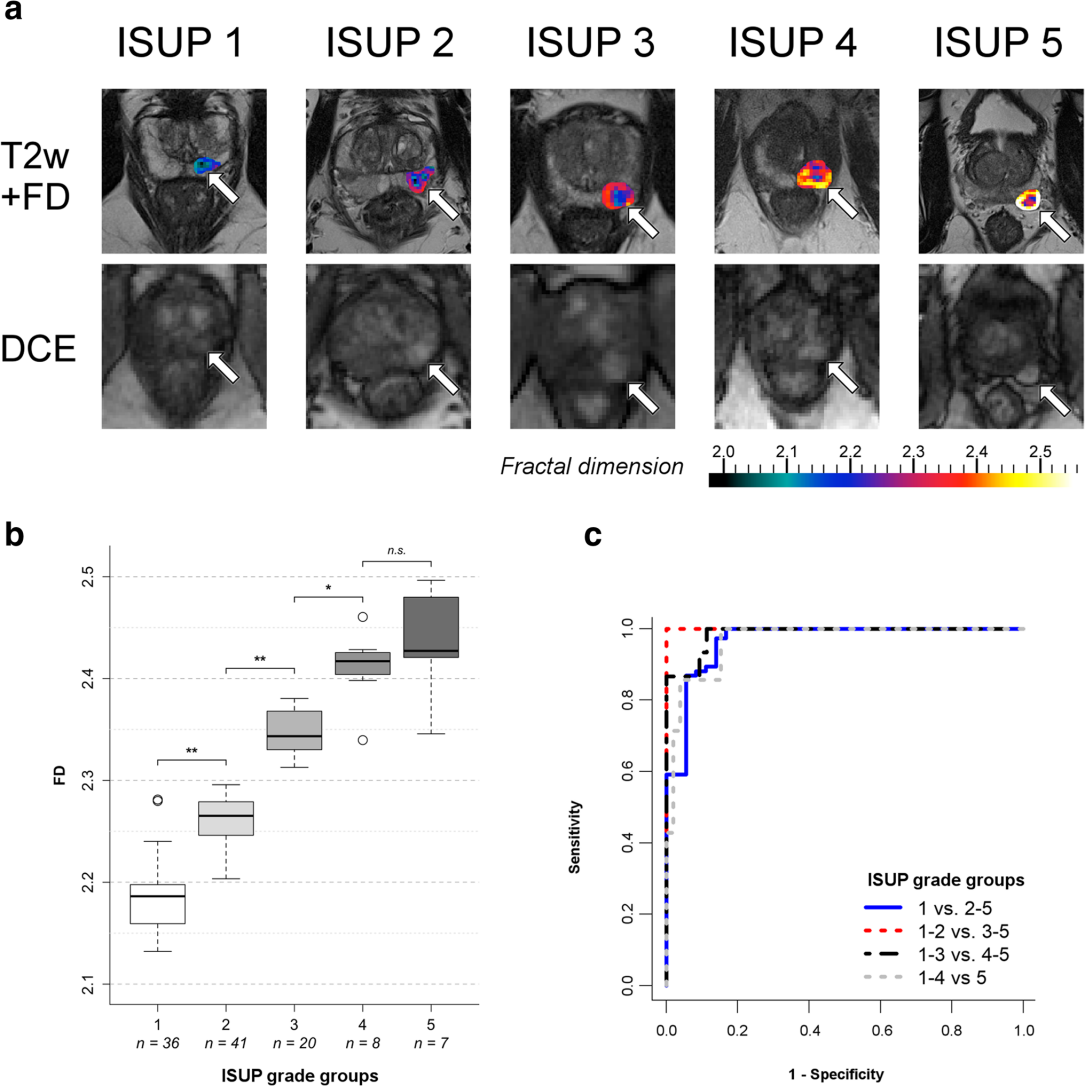
Application of fractal analysis to clinical MR imaging data of prostate cancer. **a** One representative example of fractal analysis of prostate cancer perfusion for each ISUP grade group is shown. All cancers are similar in morphologic appearance and location, i.e., left peripheral zone (arrows). The first row shows color-coded maps of the local fractal dimension (FD) of perfusion in the whole tumor fused with T2-weighted MR images (T2w + FD) for anatomic correlation. Note that the margin of the tumor, which is considered to be pathophysiologically relevant, is clearly depicted and can be differentiated by its FD from the tumor core. The second row shows the corresponding dynamic contrast-enhanced (DCE) MR images, which constitute the input for fractal analysis. **b** Boxplot of fractal dimension (FD) categorized by ISUP grade group. Significances of groupwise differences are indicated by asterisks. **c** Receiver-operating characteristic curves of fractal dimension (FD) for the differentiation of prostate cancers in dichotomized pooled ISUP grade groups. Sensitivity was defined as the fraction of correctly identified lesions in the higher-grade group pool. *—*p* = .001; **—*p* < .001; n.s.—not significant; *n*—sample size per group; ISUP grade group 1—Gleason score ≤ 6; group 2—Gleason score 3 + 4 = 7; group 3—Gleason score 4 + 3 = 7; group 4—Gleason score 4 + 4 = 8; 3 + 5 = 8; 5 + 3 = 8; group 5—Gleason scores 9–10

Fractal analysis was applied to prostate perfusion MRI sequences in 99 patients with a total of 112 prostate cancer lesions (including 16 lesions in the transitional zone). Median patient age was 65 years (range 42–78 years) with median PSA level of 12 ng/ml (interquartile range, IQR: 10.3 ng/ml) and median lesion size of 17 mm (IQR: 8.5 mm); see also [29]. The ISUP grade group distribution can be found in Fig. 3. Fractal analysis was successful in all lesions and took approximately 10 min per lesion including preprocessing, calculation of fractal dimension (FD) maps, definition of regions of interest (tumor margin), and evaluation of the fractal analysis result. Examples of fractal analysis in one exemplary prostate cancer for each of the five ISUP grade groups are depicted in Fig. 3a.

Figure 3b shows a boxplot of the correlation of FD with ISUP grade groups including sample size per group with significances of pairwise multiple group comparisons for differences in median FD. The Kruskal–Wallis test showed highly significant differences between groups (*p* < 0.001), which were identified in pairwise multiple group comparisons between low-grade (group 1), intermediate-grade (group 2), and high-grade (groups 3 and 4) lesions (Fig. 3b). No significant difference was found between the two highest tumor grade lesions (group 4, *n* = 8, versus group 5, *n* = 7). The lesionwise FD showed a strong positive linear correlation to grade group (*r*^2^ = 0.874, *p* < 0.001), and FD distribution had a spectrum-like appearance. Interobserver agreement was high with Cohen’s *κ* = 0.91 (CI: 0.85–0.97), and Bland–Altman analysis showed no substantial bias (0.016, CI: 0.01–0.023) and acceptable limits of agreement (− 0.031 to 0.063) in a subset of *n* = 50 cases.

Results of ROC analyses with AUC, sensitivity, and specificity for the dichotomized dataset with pooled ISUP grade groups are given in Table 1. ROC curves for the differentiation of lesions in pooled grade groups are given in Fig. 3c. Diagnostic accuracy for differentiating pooled lower- and higher-grade lesions at different grade group thresholds was very good (see Table 1 for all pooled group analyses including 95% confidence intervals). AUC for individual multiclass differentiation was 0.95 (95% confidence interval [CI]: 0.91–0.99). FD cutoffs for differentiating ISUP grade groups can be found in Table 2.

**Table 1:** Results of the discovery study. Receiver-operating characteristic (ROC) analysis was performed for two-class prediction by dichotomizing the dataset as well as multiclass prediction of pooled ISUP grade groups; 95% confidence intervals (CI) are given in brackets

Pooled ISUP grade group comparison	AUC	Sensitivity	Specificity
Fractal dimension			
1 versus 2–5	0.97 (CI: 0.93–1.0)	91% (CI: 83–96%) 69/76	86% (CI: 73–94%) 31/36
1–2 versus 3–5	1.0 (CI: 0.97–1.0)	100% (CI: 92–100%) 35/35	100% (CI: 96–100%) 77/77
1–3 versus 4–5	0.99 (CI: 0.97–1.0)	87% (CI: 64–98%) 13/15	100% (CI: 97–100%) 97/97
1–4 versus 5	0.97 (CI: 0.92–1.0)	43% (CI: 13–77%) 3/7	99% (CI: 96–100%) 104/105
Multiclass prediction	0.95 (CI: 0.91–0.99)	–	–
Apparent diffusion coefficient			
1 versus 2–5	0.77 (CI: 0.67–0.88)	91% (CI: 83–96%) 69/76	61% (CI: 43–77%) 22/36

**Table 2:** Cutoff values of fractal dimension (FD) for differentiation of pooled ISUP grade groups, determined as efficiency cutoffs as described under “Materials and methods”

Pooled ISUP grade group comparison	FD cutoff
1 versus 2–5	2.20 (*p* < .001)
1–2 versus 3–5	2.31 (*p* < .001)
1–3 versus 4–5	2.40 (*p* = .001)

Fractal analysis performed significantly better in predicting clinically significant cancer as ADC measurements (AUC_FD_ = 0.97 [CI: 0.93–1.0] versus AUC_ADC_ = 0.77 [CI: 0.67–0.88], *p* < 0.001). As shown in Table 1, ADC differentiated only ISUP grade group 1 from pooled grade groups 2–5 lesions, whereas fractal analysis accurately differentiated individual grade groups except for group 5 lesions.

## Discussion

This study introduces the fractal dimension of perfusion patterns in the margin of prostate carcinoma as a pathophysiologically meaningful measure. As such, fractal dimension has very good diagnostic accuracy for predicting the ISUP grade group. Most notably, an excellent differentiation among low- to intermediate- and high-grade lesions (corresponding to grade groups 1–4) is achieved, which implies important prognostic relevance and might be used to guide clinical management.

According to the observations in this study, fractal dimension shows a continuous distribution with a comparatively large range. This suggests the hypothesis that the development of PCa is a continuous spectrum and that the Gleason grading system, which is used in clinical practice, is merely a discretized and simplified, thus clinically seizable, approximation to tumor biology. This perspective gains relevance with regard to the WHO’s recent recommendation to report the fraction of Gleason grade 4 and to consider the reporting of the Gleason grade 5 fraction [15]. On the one hand, the precise characterization in intermediate-grade lesions has important prognostic implications [39–41]. On the other hand, patients with ISUP grade group 2 lesions and a low Gleason grade 4 fraction might be eligible to active surveillance [16]. The Gleason grading system has traditionally been used for clinical decision-making and constitutes a decent estimator for prognosis. However, along with more elaborate imaging techniques, see, e.g. [42, 43], and clinical evidence of diagnostic performance of multiparametric MRI [17, 18] by implementing PI-RADS version 2.1 [21], the need for a pathophysiologically more elaborate approach to PCa grade prediction arises. Moreover, along with a continuing trend towards ever-shorter acquisition protocols, e.g., in [44], no reliable method to non-invasively predict PCa grading has yet been implemented in the clinic. Fractal analysis of perfusion is an auspicious candidate for this challenge and constitutes a means of analysis which might turn DCE sequences to our advantage.

From a clinical perspective, fractal analysis can be straightforwardly implemented into clinical routine, if a DCE imaging sequence was performed, since it does not require any particular imaging prerequisites. FD might be added to PI-RADS assessment as an additional quantitative criterion: The potentially added informative value of fractal analysis in lesions with intermediate probability of malignancy (i.e., PI-RADS 3) might be to identify clinically non-significant (ISUP group 1) PCa to stratify biopsy priority and method, or to justify active surveillance. Moreover, changes in FD might indicate cancer progression in active surveillance patients. In PI-RADS 4 and 5 lesions, fractal analysis might support clinical decision-making by differentiating high-grade from low-grade lesions according to ISUP group.

The fractal organization of PCa biology has been scrutinized in previous research and involves a cytology [45, 46], a vascular morphology [47, 48], and an imaging feature (T2-weighted images for tumor detection) perspective [49, 50]. In a recent study with implanted tumor cells into mice, fractal analysis of images obtained after administration of an intravascular contrast agent allowed non-invasive characterization of microvascular architecture [48]. This aspect gains clinical importance given the fact that the blood vessel tree morphology has been shown to be predictive for mortality due to its relevance for tumor progression and metastatic potential [51]. Moreover, perfusion MRI as a functional imaging method has shown the potential to yield pathophysiologically relevant information, e.g., for the assessment of aggressiveness of PCa in the peripheral zone [23].

In the context of PCa, the margin of the hypervascularized part of the tumor is an optimal target for fractal analysis due to its decisive role in angiogenesis and tumor development. Angiogenesis is governed by (patho)physiology-determined boundary conditions. These boundary conditions arise from local and global growth stimuli and are promoted by carcinoma cells of an angiogenic phenotype [52]. The structural and functional characteristics of the blood vessel tree implicate distinct perfusion patterns which constitutes the pathophysiological justification for the hypothesis in this study. One scope of this study is to provide the proof of concept of fractal analysis of perfusion imaging in PCa. As such, it shows that fractal dimension intrinsically features pathophysiological implications in PCa and is readily implemented into standard DCE sequences. Moreover, a validation study in a separate population has independently established diagnostic accuracy of the established FD cutoffs [53].

Other than DCE imaging, several techniques have been proposed for perfusion imaging, most notably, intravoxel incoherent motion (IVIM) [54] and arterial spin labeling (ASL) [55]. To improve understanding of perfusion characteristics, potentially even beyond the tumor transition zone, and to enable perfusion imaging without the need for Gd-based contrast agents, further research might evaluate the application of fractal analysis to those imaging techniques.

The following challenges and limitations need to be discussed. The in silico phantoms generated in this study systematically vary one aspect of tumor vessel morphology, i.e., the optimization target of the vascular tree. However, with further progressive dedifferentiation, the impact of vascular optimization is thought to diminish. Rather, extreme metabolic and mechanical properties gain importance in higher tumor grades. Therefore, the model and phantoms presented depict early aspects of the process of tumor angiogenesis and hardly provide a comprehensive model of the complex process of tumor angiogenesis, especially in highly dedifferentiated tumors. However, from a clinical perspective and for prostate cancer in particular, the discrimination of lower and intermediate tumor grades without metastatic spread is most relevant for therapeutic management and therefore justifies the chosen approach. The trees feature pseudorandomly distributed terminal locations, which is unlikely to occur in tumor tissue. The generation algorithm of the trees assumes laminar and steady flow, which is justified for medium- to small-sized vessels, in which steady-state flow dominates over oscillatory flow [56]. Cancer in the transitional zone of the prostate is a major diagnostic challenge due to the heterogeneous tissue architecture of this zone, especially in men with hyperperfused nodules of benign prostatic hyperplasia, which is a common condition.

From a total of 112 lesions, only eight lesions with ISUP grade group 4 and seven lesions with group 5 were available in the dataset. This small sample size does not allow concluding on the diagnostic accuracy of fractal analysis in the differentiation between those highest-grade lesions. Moreover, the number of tumors in the transitional zone was small (*n* = 16), which does not allow for a meaningful subgroup analysis per region.

Also, clinical information about the patients like stage, clinical management, or follow-up data were not included in the dataset. No differentiation of the histological entity of PCa was available. However, the introduction of intraductal carcinoma as a new entity of PCa and new variants of acinar PCa into the classification of tumors by the WHO [15] is not adequately reflected in the analyzed dataset. Another important aspect is tumor tissue sparsity, with tumor tissue being intermixed with normal prostate tissue. Those tumors are specifically depicted in DCE images, and differentiation to prostatitis can be challenging [57]. The intermixture of normal prostate tissue might alter the perfusion pattern, thus affecting results of fractal analysis. It might be assumed that histological characteristics such as tumor entity and tumor sparsity have implications on prognosis or diagnostic accuracy, respectively. Therefore, dedicated fractal analysis of perfusion according to histological tumor characteristics might be insightful. The available reference standard from the dataset was in-bore MRI-guided biopsy. In comparison to radical prostatectomy, a sampling bias due to biopsy location might be present. However, results from in-bore MRI-guided biopsy have shown 88% correspondence with Gleason grading from radical prostatectomy [58]. Therefore, this biopsy method is considered a “problem-solver” due to its high accuracy including difficult and small cancer lesions [59].

In summary, fractal analysis of perfusion territories is a meaningful concept of pathophysiological perfusion imaging. An algebraic formulation of fractal perfusion territories is reported in this work, which is validated using in silico phantoms. The application of fractal analysis to clinical MR imaging data of prostate cancer in this study has shown high potential for non-invasive grade prediction of prostate cancer. From a translational perspective, fractal analysis could be easily implemented into current mpMRI of prostate cancer, e.g., in terms of a separate PI-RADS criterion, which is an excellent tool for cancer detection but so far lacks the ability to accurately predict tumor grade. Fractal dimension—a quantitative measure of geometrical chaos—is proposed as a meaningful imaging biomarker with distinctive pathophysiological significance for oncological diagnosis using a non-invasive imaging test.

## Supplementary Information

Below is the link to the electronic supplementary material.

Supplementary file1 (DOCX 19516 kb)

## Abbreviations

DCE: Dynamic contrast-enhanced
FD: Fractal dimension
ISUP: International Society of Urological Pathology
mpMRI: Multiparametric MRI
PCa: Prostate carcinoma

## Declarations

### Guarantor

The scientific guarantor of this publication is Professor Marc Dewey.

### Statistics and Biometry

Alejandra Rodríguez Sánchez kindly provided statistical advice for this manuscript.

### Methodology

• retrospective

• experimental

• data from one center, analysis at two centers
