# Supplementary material for: Prediction of prostate cancer grade using fractal analysis of perfusion MRI: retrospective proof-of-principle study
Source: Eur Radiol. 2021 Dec 16;32(5):3236–47. doi: 10.1007/s00330-021-08394-8 (PMC9038862; doi:10.1007/s00330-021-08394-8)
Supplement: Supplementary file 1 — Supplementary file1 (DOCX 19516 kb) [file 330_2021_8394_MOESM1_ESM.docx]

**Supplementary Methods**

*Pathophysiological motivation for in silico experiments*

Tumor growth largely depends on angiogenesis with an ‘angiogenic switch’ as a crucial prerequisite for growth beyond a certain volume. Interaction of the tumor with its microenvironment is a major factor triggering and governing angiogenesis [1]. Angiogenesis of noncancerous normal tissue is stringently regulated and follows the principle of using a minimum of energy for creation, perfusion and maintenance of the vascular network [2; 3]. This includes the principle of ensuring efficient perfusion with a minimum of vascular volume within the tissue. Demands on vasculature change with tumor progression and, in prostate cancer, changes in vascular architecture are especially characteristical in the tumor margin [4]. Excessive formation of new vessels requires a large supply of cellular material, i.e., endothelial cells, which receive stimuli from viable angiogenesis-promoting tumor parts. With disruption of an orderly angiogenic structure, there is a shift to a vessel tree with a minimized endothelial surface at an early stage of dedifferentiation. In further course of dedifferentiation, local metabolic and mechanical conditions lead to remodeling of the vessel tree towards minimal vascular length, governed by gradients of proangiogenic factors [1]. With further progression, a myriad of biophysical stimuli lead to structural vessel abnormalities including the formation of alternative blood channels through vasculogenic mimicry [5] and lymphatic remodeling [6]. This stage involves yet incompletely understood design principles of tumor vasculature. Finally, at the most advanced stage of dedifferentiation, the tumor vessel tree hardly provides adequate and consistent perfusion. At this stage, the tumor contains focal ischemic areas with high oxidative stress, low pH and finally necrosis, which may lead to further increase in perfusion heterogeneity.

*Formulation of the pathophysiological framework*

Perfusion regulation is mainly governed by vascular levels or scales ranging from small arteries to precapillary arterioles. Since the vascular tree has fractal properties within the biological limits of scale (see animated version of **Supplementary Figure** **S1**), perfusion territories, which arise from vascular anatomy, are hypothesized to be fractal as well. In fractals, a power law relationship exists between scale and a measured variable:

$\varepsilon^{\mathrm{FD}}\propto L$ (1)

where *ε* denotes scale, *L* the measured variable and *FD* the fractal dimension. When a two-dimensional model of perfusion is used, a perfused tissue portion is represented by a certain area *A_ε_* which is defined by its dependent terminal sites as it is part of the total perfusion area *A_tot_*. Scale *ε* can therefore be defined as the inverse of the relative tissue portion, *A_perf_*:

$\varepsilon=\frac{1}{A_{\mathrm{perf}}}=\frac{A_{\mathrm{tot}}}{A_{\varepsilon}}$ (2)

Perfusion in the scale-dependent perfusion territory *Q_perf_* is governed by the respective proximal volume of the vessel tree, *V_prox_*_,_ and a corresponding time delay *t_ε_* at a given total perfusion rate *Q_tot_*:

$L=Q_{\mathrm{perf}}=\frac{V_{\mathrm{prox}}}{t_{\varepsilon} \cdot Q_{\mathrm{tot}}}$ (3)

Inserting equation (2) and (3) into (1) yields

$A_{perf}^{-FD}\propto Q_{perf}$ (4)

and represents the power law relationship between the proximal, regulating part of the vascular tree and the distal, regulated tissue portion, i.e., the perfusion territory. Hence:

$FD=\lim_{\varepsilon\to0} \frac{\ln Q_{\mathrm{perf}}}{\ln A_{\mathrm{perf}}}$ (5)

The linear relationship in a tree model is presented in the animation **Supplementary Figure** **S1**.

*Generation of vascular tree models*

The constructive constrained optimization algorithm (CCO) [7] was used to generate trees representing host and tumor vasculature. The host trees perfused a circular area of physiological, nontumorous tissue, and included a placeholder area for insertion of tumor vascular trees. Host trees used minimal vascular volume as the optimization target. Tumor trees were emulated at different stages of dedifferentiation by altering the optimization target: In higher grades of tumor dedifferentiation, minimizing mechanical perfusion costs is less relevant, as it is the case in physiological trees, which approximate optimization for intravascular volume. Therefore, increasing vascular dedifferentiation was modelled with decreasing impact of the vessel radius on the optimization cost function:

$T\left( \lambda\right)=\sum_{i=1}^{N_{tot}} l_{i}\cdot r_{i}^{\lambda}$ (9)

where *T* is the optimization target, *l* the vessel length and *r* the vessel radius. This results in three distinct stages of dedifferentiation, when using different values for *λ*: 2 (representing vascular volume), 1 (representing endothelial surface) and 0 (representing vascular length).

Five pseudorandomly produced sets of terminal branch locations were generated and three trees were calculated for each of the five sets, resulting in a total of 15 tumor trees (see **Supplementary Figures** **S2-4**). Each of the three trees per set corresponds to one of the three dedifferentiation stages according to the vascular model. These tumor trees were inserted into the placeholders of the host trees, yielding five tumor-host tree models for each of the three dedifferentiation stages. All resulting trees can be found in **Supplementary Figures** **S2-4**.

Further parameters employed in the CCO were as follows: The radius of the circular perfusion area was set to 5 cm, or 2000 pixels (host) and 1.6 cm, or 640 pixels (tumor). To properly emulate prostate cancer vasculature, microvascular density, i.e., the number of capillary terminals per area, was kept constant in host tissue and tumor trees, yielding 5000 terminals in host trees and 570 terminals in tumor trees. Blood flow was set to 500 (host) and 100 (tumor) ml per sec, corresponding to perfusion rates of approximately 6.4 (host) and 12.4 (tumor) ml per sec per cm^2^, to account for the hyperperfusion in prostate cancer. Perfusion pressure was 100 mmHg, terminal pressure was 60 mmHg, and bifurcation exponent was 3.

*Generation of in silico perfusion phantoms*

Perfusion phantoms were generated from the underlying vascular anatomy as follows: First, affiliation of a specific area unit (represented by a pixel) to the corresponding regulating part of the vascular tree was determined by the finding the closest terminal segment. Thus, each vascular segment was assigned a depending perfusion territory. Second, perfusion was simulated by rendering all pixels per perfusion territory with an intensity proportional to the FD-determining quotient ln *Q_reg_* ∙ (ln *A_reg_*)^-1^ (see above) multiplied by the perfusion rate of the territory, *Q_prox_* ∙ *A_ε_*^-1^. Third and finally, these perfusion territory maps were scaled from the original resolution of 25 μm per pixel to 1.5 mm per pixel to match the resolution of clinical DCE images.

*Implementation of fractal analysis*

Local fractal analysis [8] is based on the blanket fractal dimension [9]. As outlined above, the imaging data are considered textures. The following procedures are performed for each pixel. Two blankets, one upper and one lower blanket, are constructed around the texture, being equal to it at iteration 0. The blankets are iteratively raised or lowered from the texture, thereby losing detail. The local fractal dimension is obtained from quantifying the loss of detail as a function of distance between the iteratively displaced blankets. The loss of detail is assessed in a 3x3 pixels neighborhood:

$u_{\varepsilon}\left( i,j \right)=\max\left\{ u_{\varepsilon-1}\left( i,j \right)+1,\max_{\begin{aligned} \left| \left( m,n \right)-\left( i,j \right) \right|\leq1 \end{aligned}} u_{\varepsilon-1}\left( m,n \right) \right\}$ (6)

$b_{\varepsilon}\left( i,j \right)=\min\left\{ b_{\varepsilon-1}\left( i,j \right)-1,\min_{\begin{aligned} \left| \left( m,n \right)-\left( i,j \right) \right|\leq1 \end{aligned}} b_{\varepsilon-1}\left( m,n \right) \right\}$ (7)

where *u_ε_* and *b_ε_* represent the top and bottom surfaces, *ε* the scale, i.e., the counter of iterations, and *i*, *j*, *m* and *n* are pixel coordinates. Area *A(ε)* of the blanket is determined at each iteration by the formula:

$A\left( \varepsilon\right)=\frac{\sum_{i.j} \left( u_{\varepsilon}\left( i,j \right)-b_{\varepsilon}\left( i,j \right) \right)}{2\varepsilon}$ (8)

A fractal texture yields a bi-logarithmic linear relationship with a decreasing slope for ln *A(ε)* against ln *ε*. The slope is determined from a linear fit of ln *A(ε)* against ln *ε* and the FD is calculated as *FD* = 2 - *slope*.

*Fractal analysis of clinical prostate MRI*

First, the following preprocessing steps were applied to the imaging data prior to fractal analysis. Preprocessing included a linear intensity standardization, which was performed according to muscular contrast enhancement. Signal intensity of the internal obturator muscle was determined before and after contrast administration. To this end, a ROI was placed within the internal obturator muscle and mean intensity prior to contrast arrival measured (*I_baseline_*). Analogously, maximum muscular intensity (*I_peak_*) in the DCE series from the same ROI was obtained. Subsequently, intensity was linearly standardized (*I_stn_*) according to the following formula:

*I_stn_ =* (*I_original_ - I_baseline_*) / (*I_peak_ - I_baseline_*) (10)

Intensity *I_stn_* was scaled to an appropriate range to fit fractal analysis by multiplication by five and addition of 100 for the purpose of adequate gray value visualization. Image noise (*σ_noise_*) was estimated from the standard deviation of the ROI representing precontrast signal intensity of the internal obturator muscle. A bilateral filter [10] was applied to the imaging data with a standard deviation (SD) in the spatial domain, *σ_domain_* = 0.8. SD in the intensity range was calculated as *σ_range_* = 1.64*σ_base_* to encompass the 90% limit of a Gaussian noise distribution, thus ensuring noise-level-adapted filtering. A region of interest (ROI) was defined in a standardized manner: the interface region between the hyperperfused part of the tumor as depicted in the DCE images and the adjacent tumor harboring prostate tissue was selected by fitting a serpentine-like ROI with a constant width of 3 mm. The respective ROI was propagated to the local FD map at each point in time and corrected for motion if necessary. Subsequently, an image slice location from the DCE image sequence was defined for fractal analysis. To identify the lesion, the given coordinates of the lesion center were checked against T2-weighted images, diffusion-weighted images and perfusion images. A paramedian slice location in relation to the center of the prostate cancer lesion was selected to obtain a representative depiction of the tumor margin. From this slice location, two-dimensional maps of the local FD were calculated from the DCE image sequence for each point in time using a 3x3 pixel kernel. The mean FD of the ROI was calculated and plotted over time. The highest mean FD in the time sequence was extracted. Fractal analysis was performed without knowledge of individual ISUP grade groups.

**Supplementary Tables**

**Supplementary Table 1**. Imaging protocol details. DCE – dynamic contrast enhanced, n.a. – not applicable

| **Parameter** | **T2-weighted sequence** | **DWI sequence** | **ADC sequence** | **DCE sequence (T1-weighted)** |
| --- | --- | --- | --- | --- |
| Field strength (all sequences) | 3 Tesla | | | |
| Coil (all sequences) | pelvic phased-array coil without endorectal coil | | | |
| Fat suppression | no | | | |
| Pulse sequence | 2D turbo spin echo | single-shot echo-planar imaging (three directions) | secondary calculation | 3D turbo flash gradient echo |
| Orientations | axial, sagittal, coronal | axial | axial | axial |
| Resolution (mm) | ~ 0.5 x 0.5 | 2 x 2 | 2 x 2 | 1.5 x 1.5 |
| Slice thickness (mm) | 3.6 | 3.6 | 3.6 | 4 |
| Field of view (mm) | 180 x 180 –  192 x 192 | 168 x 256 | 168 x 256 | 192 x 192 |
| Temporal resolution | n.a. | n.a. | n.a. | every 3.5 s for  2:40 min |
| b-values (s∕mm^2^) | n.a. | measured: 50, 400, 800  calculated:  1400 | n.a. | n.a. |

**Supplementary Figures**

**
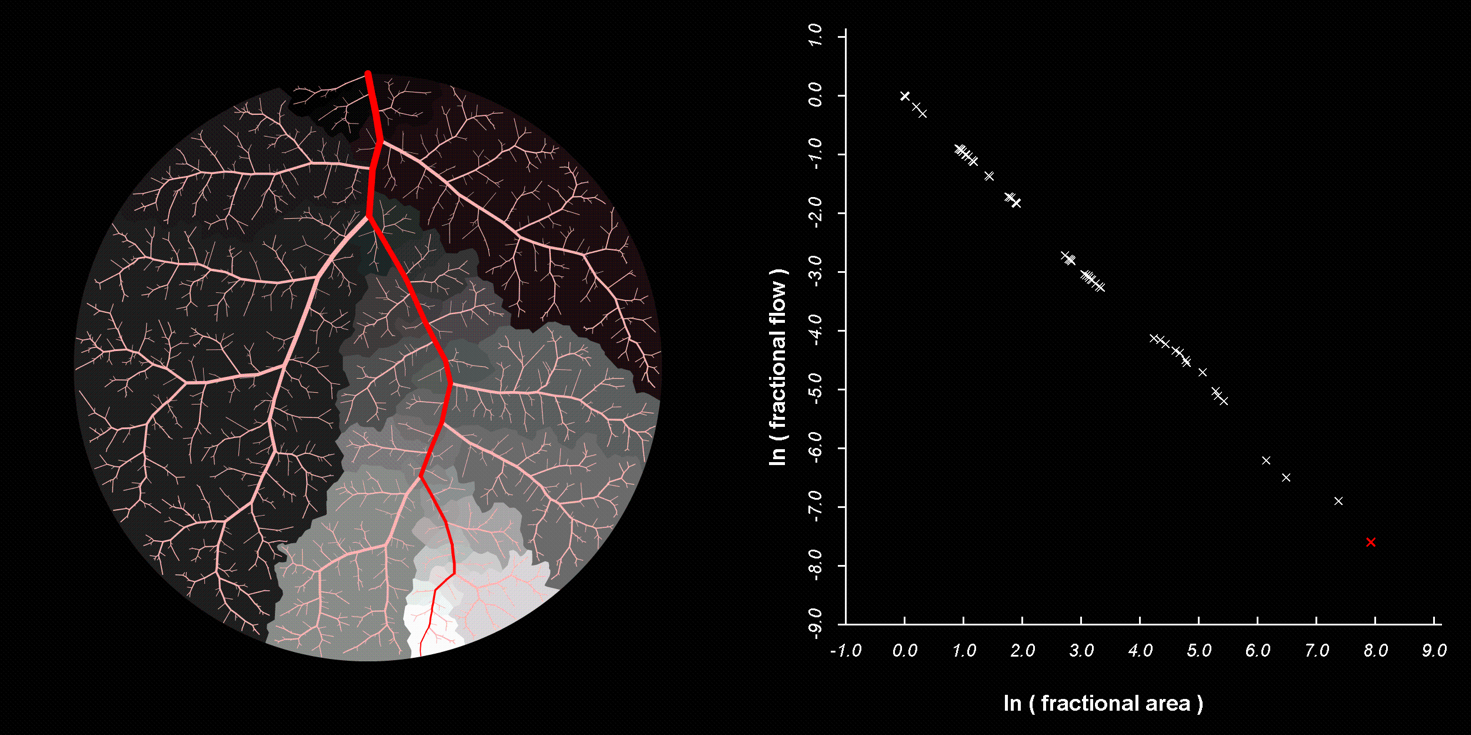
**

**Supplementary Figure S1.** *(See separate GIF animation below)* Animation illustrating the fractal relationship between flow and perfusion territories.

This is the animated version of the perfusion territories shown in Figure 1A in the main manuscript. The proximal part of the vascular tree (red) and the depending vascular tree (blue) and perfusion territory (gray) are displayed for each vascular scale in one exemplary branch. The corresponding measures of ln *Q_reg_* and ln *A_reg_* are indicated in the diagram. The fractal organization of flow and perfusion territory is indicated by a linear bi-logarithmic relationship in the graph. This relationship is the pathophysiological justification for the concept developed in this study and is intrinsically independent of a specific imaging application. For clarity of presentation, vascular trees were created with 3000 terminal sites but without placeholders for tumor vascular architecture.


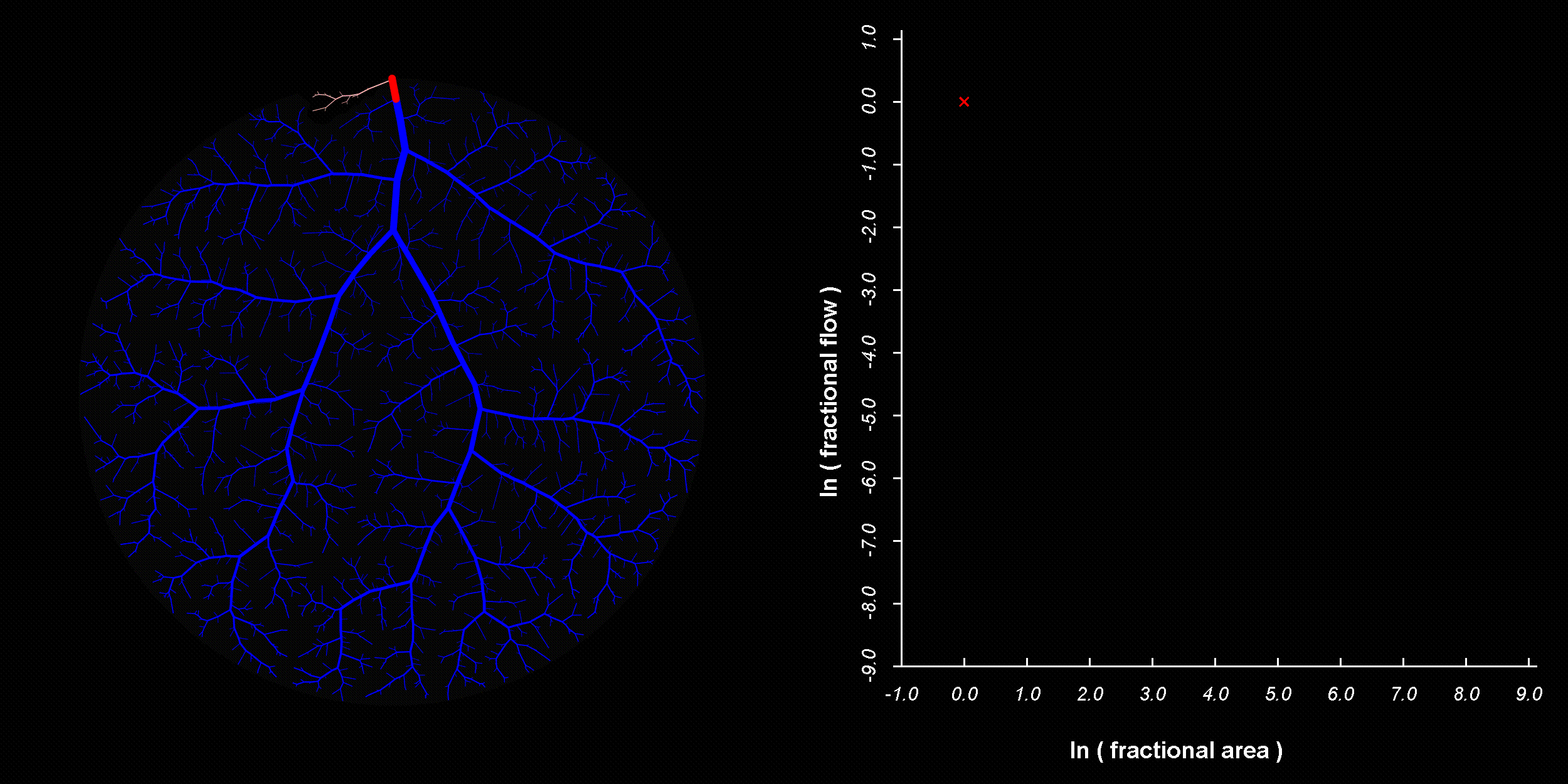


**
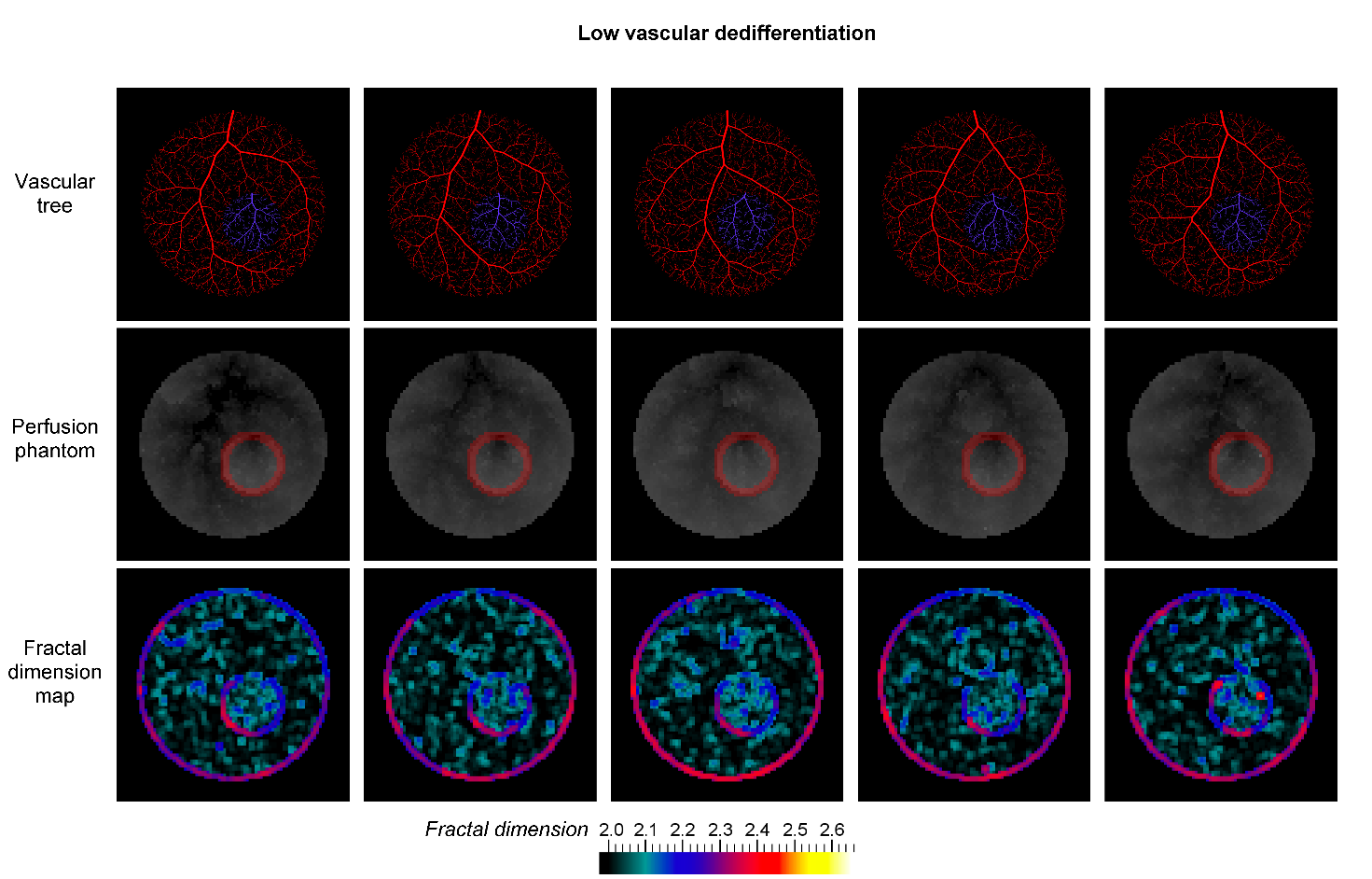
**

**Supplementary Figure S2.** Simulation of low vascular dedifferentiation.

**
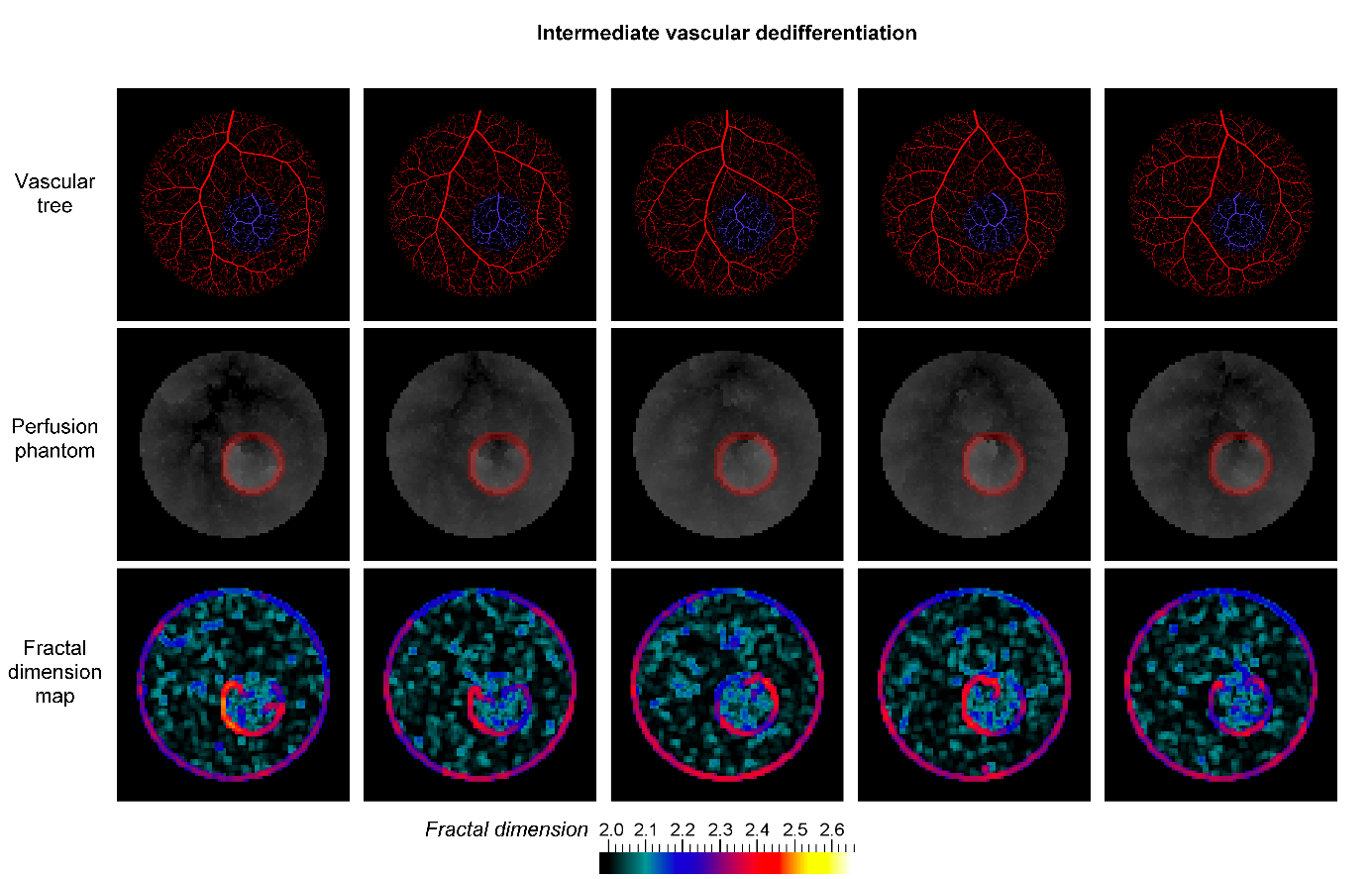
**

**Supplementary Figure S3.** Simulation of intermediate vascular dedifferentiation.

**
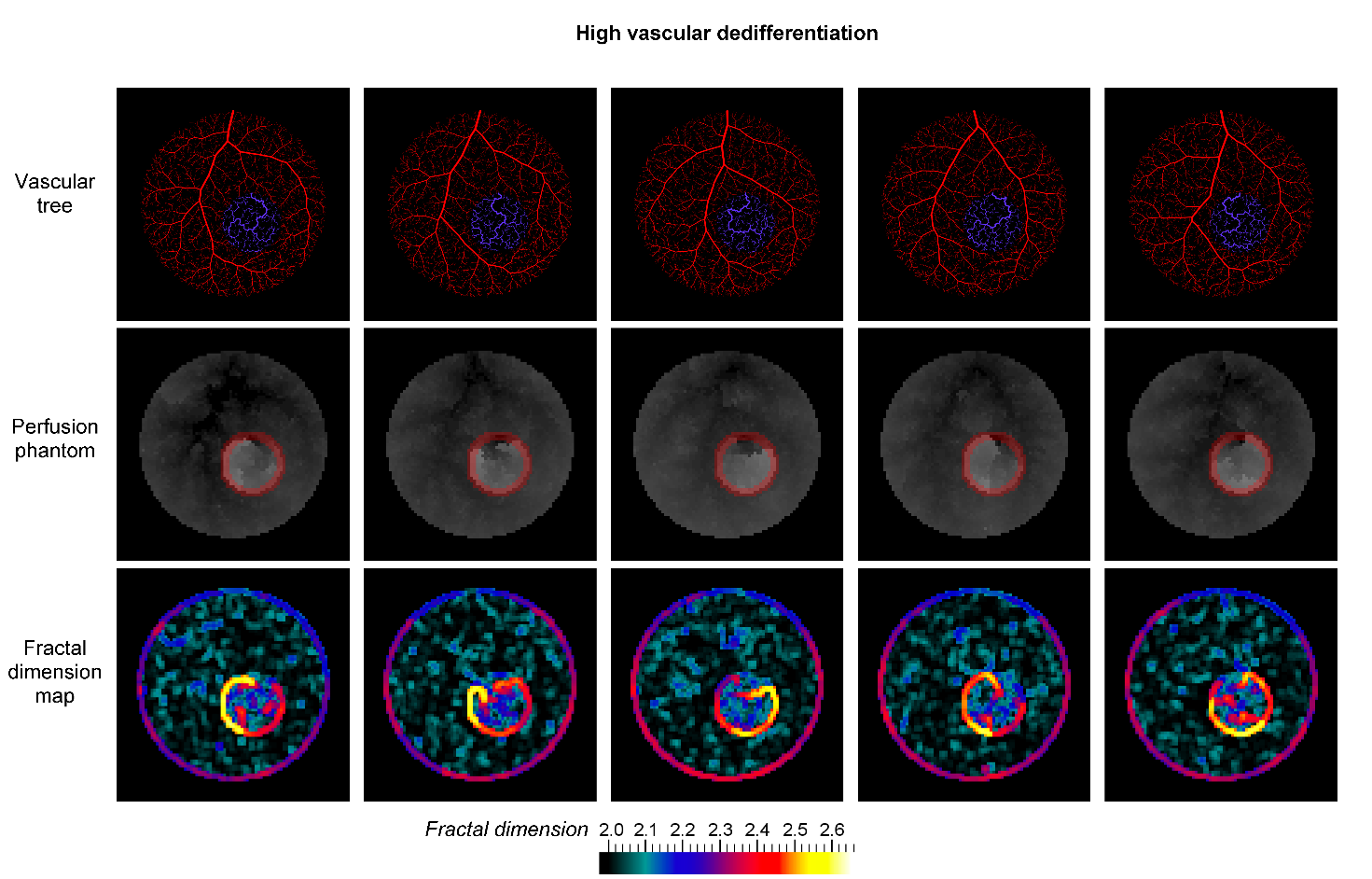
**

**Supplementary Figure S4.** Simulation of high vascular dedifferentiation.

For each dedifferentiation grade (low, intermediate, high), vascular trees consisting of a host tree (red) and a tumor tree (purple) were created. The host trees were the same for all groups. The tumor trees for all three staged (grouped in separate figures and sharing the same left-to-right arrangement) shared the same pseudorandomly generated terminal site locations (top row). Perfusion territories (middle row) were calculated and scaled to match clinical resolution (1.5 mm per pixel); red indicates the interface region of host and tumor perfusion. The fractal dimension (FD) was extracted from the interface region (red) on the perfusion phantoms. The same top-to-bottom arrangement of the panels as in Figure 3A in the main manuscript is used here, and the five phantoms for each of the three dedifferentiation stages are presented in separate Supplementary Figures S2-4.

**Supplementary References**

1 Weis SM, Cheresh DA (2011) Tumor angiogenesis: molecular pathways and therapeutic targets. Nat Med 17:1359-1370

2 Kamiya A, Togawa T (1972) Optimal branching structure of the vascular tree. Bull Math Biophys 34:431-438

3 Kassab GS (2006) Scaling laws of vascular trees: of form and function. Am J Physiol Heart Circ Physiol 290:H894-903

4 Tilki D, Seitz M, Singer BB et al (2009) Molecular imaging of tumor blood vessels in prostate cancer. Anticancer Res 29:1823-1829

5 Wang H, Lin H, Pan J et al (2016) Vasculogenic Mimicry in Prostate Cancer: The Roles of EphA2 and PI3K. J Cancer 7:1114-1124

6 Ruoslahti E (2002) Specialization of tumour vasculature. Nat Rev Cancer 2:83-90

7 Schreiner W (2001) Concepts and features of arterial tree models generated by constrained constructive optimization. Comments Theor Biol 6:103-136

8 Novianto S, Suzuki Y, Maeda J (2003) Near optimum estimation of local fractal dimension for image segmentation. Pattern Recognition Letters 24:365-374

9 Peleg S, Naor J, Hartley R, Avnir D (1984) Multiple resolution texture analysis and classification. IEEE Trans Pattern Anal Mach Intell 6:518-523

10 Tomasi C, Manduchi R (1998) Bilateral filtering for gray and color images. In: Computer Vision, 1998. Sixth International Conference on, 4-7 Jan 1998. 839-846.
